# Expanding Geriatric Education Across the University and Country: Schaalman Senior Voices Program

**DOI:** 10.1093/geroni/igaf122.4394

**Published:** 2025-12-31

**Authors:** Erin Emery-Tiburcio, Grisel Rodriguez-Morales, Laurin Mack, Franco Sambataro, Robyn Golden

**Affiliations:** Rush University Medical Center, Chicago, Illinois, United States; Rush University Medical Center, Chicago, Illinois, United States; Rush University Medical Center, Chicago, Illinois, United States; Rush University Medical Center, Chicago, Illinois, United States; Rush University Medical Center, Chicago, Illinois, United States

## Abstract

The Schaalman Senior Voices (SSV) Program aims to expand awareness of the 4Ms of an Age-Friendly Health System across Rush University, and around the country. This aim was achieved by offering: student fellowships, in which health professions students complete a mentored project focusing on the 4Ms; faculty fellowships, in which health professions faculty develop and integrate the 4Ms into existing curriculum; a six-session, twelve-hour Summer Academy on Aging for Rush University students and staff; and a Thought Leader Lectureship in which a leader in the aging network presents to a national audience. In the 2024-2025 academic year, three student fellows, three faculty fellows, 19 Summer Academy students, and one thought leader with 269 attendees participated in Schaalman activities. Outcomes of student fellowships included significant increases in awareness of audiology services among primary care staff, development of a training module for direct care workers on bathing and toileting within the 4Ms framework, and increased knowledge about delirium among nursing staff. Faculty fellows created unique, interactive 4Ms curriculum within an anesthesiology clerkship, an undergraduate pathophysiology course, and occupational therapy assessment training. Summer Academy learners demonstrated an average of 21.27% increase in knowledge (range 4.41%-65.7% increase). Outcomes of the Thought Leader Lectures included (n = 241 responses) 98% satisfaction with content and 96% found the content directly applicable to practice. Implementation of the SSV project has significantly increased access to aging education and taken great strides in creating the 4Ms as foundational learning across Rush University colleges. Implications for policy and dissemination will be discussed.

